# A Two-Part Mixed Model for Differential Expression Analysis in Single-Cell High-Throughput Gene Expression Data

**DOI:** 10.3390/genes13020377

**Published:** 2022-02-18

**Authors:** Yang Shi, Ji-Hyun Lee, Huining Kang, Hui Jiang

**Affiliations:** 1 Division of Biostatistics and Data Science, Department of Population Health Sciences and Department of Neuroscience and Regenerative Medicine, Medical College of Georgia, Augusta University, Augusta, GA 30912, USA; yshi@augusta.edu; 2 Department of Internal Medicine, University of New Mexico Comprehensive Cancer Center, Albuquerque, NM 87102, USA; 3 Department of Biostatistics, University of Michigan, Ann Arbor, MI 48109, USA; 4 Division of Quantitative Sciences, University of Florida Health Cancer Center and Department of Biostatistics, University of Florida, Gainesville, FL 32610, USA; jihyun.lee@ufl.edu; 5 Department of Internal Medicine, University of New Mexico, Albuquerque, NM 87131, USA; 6 Center for Computational Medicine and Bioinformatics, University of Michigan, Ann Arbor, MI 48109, USA; 7 University of Michigan Rogel Cancer Center, University of Michigan, Ann Arbor, MI 48109, USA

**Keywords:** two-part mixed-model, single-cell RNA-seq, single-cell RT-qPCR, differential expression, automatic differentiation

## Abstract

The high-throughput gene expression data generated from recent single-cell RNA sequencing (scRNA-seq) and parallel single-cell reverse transcription quantitative real-time PCR (scRT-qPCR) technologies enable biologists to study the function of transcriptome at the level of individual cells. Compared with bulk RNA-seq and RT-qPCR gene expression data, single-cell data show notable distinct features, including excessive zero expression values, high variability, and clustered design. We propose to model single-cell high-throughput gene expression data using a two-part mixed model, which not only adequately accounts for the aforementioned features of single-cell expression data but also provides the flexibility of adjusting for covariates. An efficient computational algorithm, automatic differentiation, is used for estimating the model parameters. Compared with existing methods, our approach shows improved power for detecting differential expressed genes in single-cell high-throughput gene expression data.

## 1. Introduction

Recently, single-cell high-throughput gene expression profiling technologies, including single-cell RNA sequencing (scRNA-seq) and parallel single-cell single-cell reverse transcription quantitative real-time PCR (scRT-qPCR), have enabled researchers to examine mRNA expression at the resolution of individual cell level, which provide further biological insights of the transcriptomes and functional genomics [1,2,3,4]. Compared to bulk RNA-seq and RT-qPCR experiments that are usually performed on animal tissues (i.e., cell populations) and homogenous cell lines, single-cell high-throughput gene expression data generated by scRNA-seq and scRT-qPCR have the following distinct features as seen in recent literature [4,5,6]:

*Excessive zero expression values.* The proportions of genes with observed zero expression values in single-cell gene expression data are much larger than bulk RNA-seq or RT-qPCR data [4,5,6]. The reasons for this phenomenon can be either biological, such that the abundance of mRNA levels of certain transcripts are essentially low in individual cells, or can be technical, such that the extracted total amount of mRNA is low in a single cell sample [4,6].

*High variability of expression levels across samples.* It has been observed that scRNA-seq or scRT-qPCR data tend to show higher variability than bulk RNA-seq or RT-qPCR data [4,6]. This can be explained by the differences in the designs between the two: the regular bulk RNA-seq or RT-qPCR experiments are performed on the cell populations, and the gene expression levels from those experiments are averaged across all individual cells in the population, which dilutes the variability of gene expression levels among individual cells [6].

*Clustering of single-cell samples within subjects*. Another notable feature of single-cell high-throughput gene expression data is that each individual single-cell sample is randomly sampled from a higher-level cluster unit (e.g., patients, animals) [1,2,7]. Therefore, the single-cell samples from the same subject are expected to be more homogeneous than those from different subjects, which has been shown in several single-cell RNA-seq data published recently [1,2,7]. From a statistical perspective, this feature is called the clustering effect, which should be adequately adjusted for in the analysis.

To account for the abovementioned issues, we propose to model single-cell high-throughput gene expression data using a two-part mixed model. This model not only adequately accounts for the above features of single-cell gene expression data but also provides flexibility for adjusting for covariates in the study design. The details of this model and how it can be applied to differential expression analysis of single-cell data are discussed in the rest of this paper, which is organized as follows. First, we describe the formulation of the two-part mixed model with a brief literature review. Then we use an efficient method, named automatic differentiation, to fit the model. We also discuss how to test for differential expression under this model and describe several methods for approximating the null distribution of the test statistics for small sample sizes, followed by simulations for studying the type I error rate and statistical power. Finally, we demonstrate our approach by applying it to two real-world single-cell high-throughput gene expression datasets: one from scRT-qPCR and the other from scRNA-seq.

## 2. Materials and Methods

### 2.1. The Two-Part Mixed Model for Single-Cell Gene Expression Data

We first introduce the notations for our approach. Assume there are m subjects and N genes in a scRNA-seq experiment, and $n_{i}$ single-cell samples extracted and sequenced for subject (i=1,…,m). Let $y_{ijk}$ be the normalized expression value (in the unit of RPKM/FPKM, TPM, or CPM) for gene k (k=1, …,N) in single-cell sample j ($j=1,\;\ldots ,n_{i}$) in subject i, then we model the gene expression value $y_{ijk}$ using the following two-part mixed model:

(1) $$\begin{array}{l} \mathrm{logit}[\mathrm{Pr}(y_{ijk}=0)]=\log(\frac{\pi_{ijk}}{1-\pi_{ijk}})=w_{k}^{T}\alpha_{k}+u_{ik},\\ \log(y_{ijk}+c|y_{ijk}>0)=x_{k}^{T}\beta_{k}+v_{ik}+e_{ijk}, \end{array}$$

where $\pi_{ijk}$ is the proportion of single-cell samples with zero expression values for gene k (named “zero-proportion” hereafter). In this two-part model, the zero-proportions are modeled by a logistic regression model (logistic or binomial part), and the log-transformed non-zero expression values are modeled by a linear regression model (Gaussian part), where $w_{k}^{T}$ and $x_{k}^{T}$ are the vectors of covariates for the binomial and Gaussian parts, respectively (e.g., if there are only two biological conditions and no other covariates to be adjusted, $w_{k}^{T}$ and $x_{k}^{T}$ are simply the vectors of 1/0 indicators for the biological conditions), $\alpha_{k}$ and $\beta_{k}$ are the corresponding vectors of regression coefficients associated with the covariates $w_{k}^{T}$ and $x_{k}^{T}$, $e_{ijk}$ is the random error that is assumed to be distributed as $N(0,\;\sigma_{e}^{2})$, $u_{ik}$ and $v_{ik}$ are the random effects for subject i that account for the clustering effects, which are assumed to follow the bivariate normal distribution.

(2) $$\left(\begin{array}{c} u_{ik}\\ v_{ik} \end{array}\right)\sim N(0,\;\left(\begin{array}{cc} \sigma_{u}^{2} & \rho \sigma_{u}\sigma_{v}\\ \rho \sigma_{u}\sigma_{v} & \sigma_{v}^{2} \end{array}\right))$$

with $\sigma_{u}^{2}$ and $\sigma_{v}^{2}$ as the variances for the marginal univariate normal distributions of $u_{ik}$ and $v_{ik}$, and ρ as the correlation between them. We note that most scRNA-seq experiments contain only one level of clusters (i.e., single cells are sampled from subjects). If the study design is more complicated, such that it may contain multi-level cluster effects, then more variance components for the random effects can be added into the model. Finally, a small constant *c* is added to the non-zero expression levels before taking logarithms to avoid the left skewness caused by taking logarithms on small-expression values between 0 and 1, which is often seen in RNA-seq data. In the following analysis of scRNA-seq data, c is set as 1.

In an scRT-qPCR experiment, the gene expression levels are usually measured by the expression threshold (et) values, which is defined as $et=c_{\max}-ct$, where $c_{max}$ is the maximum number of amplification cycles used in the scRT-qPCR experiment and ct is the threshold cycle that the gene is detected by the PCR instrument [5]. The gene expression level $y_{ijk}$ is assumed to have an exponential relationship with et, such that $y_{ijk}=2^{et}$ (for undetected genes, et is shown as missing values from the PCR machine and can be treated as −∞, which gives zero expression values) [5]. Therefore Model (1) can also be used to model gene expression values in scRT-qPCR data, and the definitions of the parameters are exactly the same as those aforementioned for scRNA-seq data. The only difference is that adding the small constant *c* is not necessary for scRT-qPCR data, as the non-zero gene expression levels in scRT-qPCR experiments do not have many small values between 0 and 1, such as those in scRNA-seq data.

*Remark on related literature*: The two-part model including the binomial part and Gaussian part without random effects is first proposed for modeling the medical care data [8,9], where the dependent variable (medical care expenses) takes the range of any non-negative value but has a positive probability at zero (these type of data are also called semicontinuous data) [8,9,10]. This type of model is later extended for longitudinal or clustered semicontinuous data by incorporating random effects for both the binomial part and the Gaussian part [11]. A comprehensive survey for a variety of models with applications for data taking non-negative values with a substantial proportion of zero values is given in [10]. Our two-part mixed model essentially follows the model formulation in [10,11], except for the addition of a small constant *c* to the non-zero expression values in RNA-seq data [Equation (1)]. A similar yet different two-part model without random effects is proposed to model the scRNA-seq data in a recent paper, which is named MAST [12]. Instead of incorporating clustered random effects from subjects, MAST uses an empirical Bayes method to shrink the gene-specific variance to the global variance of all genes [12].

### 2.2. Model Fitting

The proposed two-part mixed model (1) will be referred to as TMM hereafter. Since the TMM is fitted for each gene independently, we will drop the subscript k for simplicity if there is no ambiguity within the context. Following [11], the fixed-effect parameters of the TMM model, $\alpha_{k}$ and $\beta_{k}$, are estimated by maximizing the following marginal likelihood function of the model:

(3) $$L\propto \prod_{i=1}^{m}\int L_{B_{i}}L_{G_{i}}p(u_{i},v_{i})du_{i}dv_{i},$$

where $L_{B_{i}}$ is the conditional distribution (likelihood) of $y_{ijk}$ given the random effect $u_{i}$ from the binomial (logistic) part that can be written as

(4) $$L_{B_{i}}=[\prod_{j=1,y_{ij}=0}^{n_{i}}\exp(w_{j}^{T}\alpha_{j}+u_{i})][\prod_{j=1}^{n_{i}}\frac{1}{1+\exp(w_{j}^{T}\alpha_{j}+u_{i})}],$$

and $L_{G_{i}}$ is the conditional distribution (likelihood) of $y_{ijk}$ given the random effect $v_{i}$ from the Gaussian part that can be written as

(5) $$L_{G_{i}}=\prod_{j=1,y_{ij}>0}^{n_{i}}\sigma_{e}^{-1}\phi [\frac{\log(y_{ij}+1)-x_{j}^{T}\beta_{j}-v_{i}}{\sigma_{e}}]$$

with ϕ(⋅) as the standard normal PDF [for scRT-qPCR data, $\log(y_{ij}+1)$ becomes $\log(y_{ij})$], and $p(u_{i},v_{i})$ is the joint distribution of the random effects $u_{i}$ and $v_{i}$, which is the bivariate normal given in Equation (2).

As discussed in [10,11], maximizing the marginal likelihood function (3) involves numerical or stochastic approximation of the integrals, followed by maximization of the approximated likelihood. Several computational methods, including the Markov chain Monte Carlo, the expectation-maximization (EM) algorithm, the penalized quasi-likelihood (PQL) method, Gauss-Hermite quadrature, and Laplace approximations are reviewed and discussed in detail in [11]. Here, we use an efficient computational method, automatic differentiation, to maximize the likelihood function (3). The automatic differentiation technique is implemented in the software package automatic differentiation model builder (ADMB, version 11.4) [13,14]. Given the likelihood function written in the form of (4.2), ADMB calculates the Hessian matrix of the marginal likelihood function using the automatic differentiation technique, and the maximization of the marginal likelihood function is performed by first approximating the integrals using Laplace approximations and then maximizing the approximated likelihood using the quasi-Newton algorithm. Descriptions of the automatic differentiation technique can be found in [13,14], and the details for implementation of the algorithm can be found in https://www.admb-project.org/ (accessed on 21 December 2021).

### 2.3. Testing for Differential Expression

Testing for differential expression of genes across biological conditions under model (1) is done by testing for the fixed effects. More explicitly, (1) can be written as

(6) $$\begin{array}{l} logit[\mathrm{Pr}(y_{ij}=0)]=\log(\frac{\pi_{ij}}{1-\pi_{ij}})=w_{1}^{T}\alpha_{1}+w_{2}^{T}\alpha_{2}+u_{i},\\ \log(y_{ij}+1|y_{ij}>0)=x_{1}^{T}\beta_{1}+x_{2}^{T}\beta_{2}+v_{i}+e_{ij}, \end{array}$$

where $w_{1}^{T}$ and $x_{1}^{T}$ are the covariates of interest that we want to test for, and $w_{2}^{T}$ and $x_{2}^{T}$ are the covariates to be adjusted for in the model. Specifically, we are interested in testing for the following two effects across biological conditions: (1) whether the zero-proportions are significantly different across conditions and (2) for genes with non-zero expression levels, whether the mean expression levels are significantly different across conditions. The two problems can be formulated as the following two corresponding hypothesis testing problems:

(1) Testing of the binomial part
  (7) $$H_{B0}:\;\alpha_{1}=0\;\mathrm{versus}\;H_{B1}:\;\alpha_{1}\neq 0;$$
(2) Testing of the Gaussian part
  (8) $$H_{G0}:\;\beta_{1}=0\;\mathrm{versus}\;H_{G1}:\;\beta_{1}\neq 0;$$
  and the two parts can also be tested jointly, which can improve the statistical power:
(3) Joint testing of the binomial and Gaussian parts
  (9) $$H_{0}:\;\alpha_{1}=0\;\mathrm{and}\;\beta_{1}=0\;\mathrm{versus}\;H_{1}:\;\alpha_{1}\neq 0\;\mathrm{or}\;\beta_{1}\neq 0.$$

The individual test for the binomial part or the Gaussian part can be performed using the Wald test or the likelihood ratio test, and the joint test for the two parts can be performed using the likelihood ratio test. Under $H_{0}$, the asymptotic distributions of the Wald statistic ($W_{0}$) and the likelihood ratio statistic ($L_{0}$) can be approximated by the $\chi^{2}$ distribution with the degrees of freedom equal to the differences in the numbers of parameters between $H_{0}$ and $H_{1}$, which is a widely used approach in practice [15,16]. However, for small sample sizes, the $\chi^{2}$ distributions are not good approximations to the null distributions of the two test statistics, which, as noted in the literature [15,17] and as shown in simulations in the Results part, often show inflated type I error rate. Therefore, we use the following two methods for reliable estimation of *p*-values when the sample size is small:

*The parametric bootstrap method*: this approach estimates the null distribution of the test statistic by simulating data from the fitted model under $H_{0}$, which is performed in the following way [17,18,19]:

(1) Fit model (4) under $H_{0}$ and generate *N* random samples $y_{1},\ldots ,y_{N}$ from this model.
(2) Calculate the corresponding test statistics (i.e., Wald or likelihood ratio statistics) $T(y_{1}),\ldots ,T(y_{N})$ using the above-simulated samples $y_{1},\ldots ,y_{N}$.
(3) Estimate the *p*-value as $\hat{p}=\frac{1}{N}\sum_{l=1}^{N}I\{T(y_{l})\geq \gamma \}$, where γ is the test statistic (Wald or likelihood ratio) calculated from the observed data (an alternative formula is $\hat{p}=\frac{\sum_{l=1}^{N}I\{T(y_{l})\geq \gamma \}+1}{N+1}$. The two formulas give almost the same results providing N is large, so we use the former throughout this chapter).

*The empirical Satterthwaitemethod*: this method is proposed in [20], and it is a general approach for approximating the null distribution of the test statistics [17,20,21,22]. Following [20,21], this method is performed in the following two steps:

(1) Approximate the null distribution of test statistics ($W_{0}$ or $L_{0}$) by a scaled $\chi^{2}$ distribution $k\chi_{v}^{2}$ with k as the scale parameter and v as the degrees of freedom. The parameters k and v can be estimated by matching the first two moments (sample mean and variance) of test statistics under $H_{0}$ with those of $k\chi_{v}^{2}$ [20,21]. The sample mean and variance of test statistics under $H_{0}$ can be obtained by using the above parametric bootstrap method with a smaller number of random samples.
(2) Fit a two-component normal mixture distribution $\pi_{1}N(\mu_{1},\sigma_{1}^{2})+\pi_{2}N(\mu_{2},\sigma_{2}^{2})$ on $\Phi^{-1}(p_{k\chi_{v}^{2}}^{\left(b\right)})$, where $p_{k\chi_{v}^{2}}^{\left(b\right)}$ is the *p*-value obtained from the above-scaled $\chi^{2}$ distribution $k\chi_{v}^{2}$ for the bth random sample and Φ(⋅) is the standard normal CDF. The final *p*-values are calculated as
  $$p=\mathrm{Pr}[\Psi >\Phi^{-1}(p_{k\chi_{v}^{2}})],$$
  where $p_{k\chi_{v}^{2}}$ is the *p*-value obtained from Step (1) and Ψ is the fitted normal mixture distribution ${\hat{\pi }}_{1}N({\hat{\mu }}_{1},{\hat{\sigma }}_{1}^{2})+{\hat{\pi }}_{2}N({\hat{\mu }}_{2},{\hat{\sigma }}_{2}^{2})$. The Satterthwaite method can estimate *p*-values using a smaller number of random samples than the parametric bootstrap method [20,21]. However, in our simulations, it also shows an inflated type I error rate when the sample size is small (see simulations in the next section).

## 3. Results

### 3.1. Simulation Studies

#### 3.1.1. Evaluation of Type I Error Rates

In this section, we evaluate type I error rates of the three methods for approximating the null distribution of the test statistics under *H*_0_: the $\chi^{2}$ distribution, the Satterthwaite method, and the parametric bootstrap method. The simulations are performed based on the following settings: assuming two biological conditions, each has m/2 subjects, and for each subject i there are $n_{i}$ single-cell samples. To evaluate type I error rates, we simulate gene expression levels $y_{ijk}$ from the following model under $H_{0}$ (i.e., there is no difference between the two conditions):

(10) $$\begin{array}{l} lgit[\mathrm{Pr}(y_{ijk}=0)]=\log(\frac{\pi_{ijk}}{1-\pi_{ijk}})=\alpha_{1}+u_{i},\\ \log(y_{ijk}+1|y_{ijk}>0)=\beta_{1}+v_{i}+e_{ij}, \end{array}$$

with $u_{i}\sim N(0,\sigma_{u}^{2})$, $v_{i}\sim N(0,\sigma_{v}^{2})$ and $e_{ijk}\sim N(0,\sigma_{e}^{2})$.

In this model, there is only one intercept for the fixed effect in both the binomial and Gaussian parts, therefore no differences in terms of zero-proportions and mean expression levels are expected between the two conditions. The values of the parameters are set as follows: $\sigma_{u}=0.5$, $\sigma_{v}=1$, $\sigma_{e}=0.5$, $\alpha_{1}\sim N(0.5,0.25^{2})$, $\beta_{1}\sim N(3,0.5^{2})$, $n_{i}=20$ for all i’s (i=1,…,m). We tune the sample sizes by varying m for 3 different values, 4, 10, and 20, respectively, which correspond to a range of increased sample sizes. The simulations are repeated 1000 times for different m’s. For each run, we calculate the following five test statistics: Wald statistic for the Gaussian part, Wald statistic for the binomial part, likelihood ratio statistic for the Gaussian part, likelihood ratio statistic for the binomial part, likelihood ratio statistic for jointly testing the Gaussian and binomial parts. Then, we calculate the *p*-values from each test using the 3 methods as described in Section 2.3.

**Figure A1:**
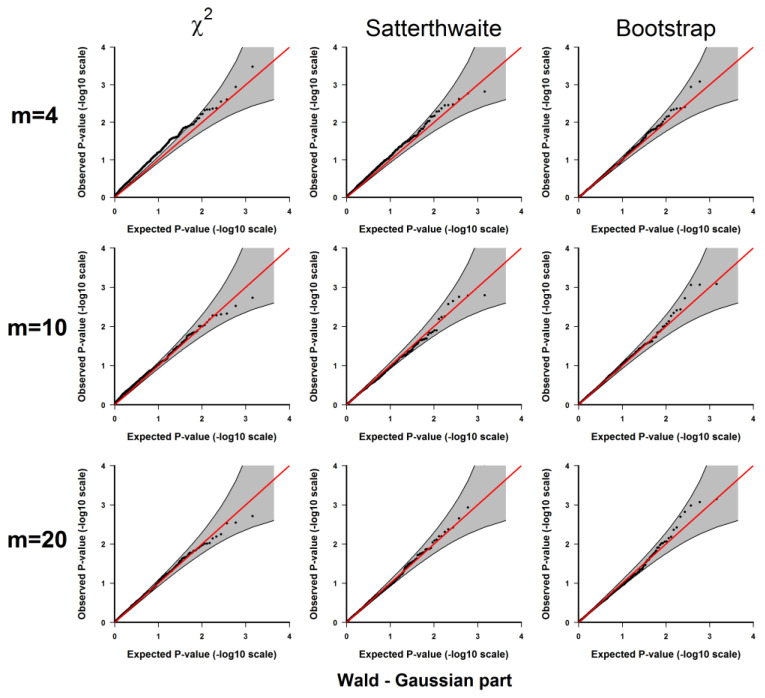
Results from 3.1.1 Evaluation of type I error rates. Plots of the observed versus the expected *p*-values for the Wald test for the Gaussian part under *H*_0_: no significant difference between the two conditions. The *p*-values are plotted on -log10 scale. The gray areas represent the 95% confidence interval bands of the expected *p*-values under *H*_0_.

**Figure A2:**
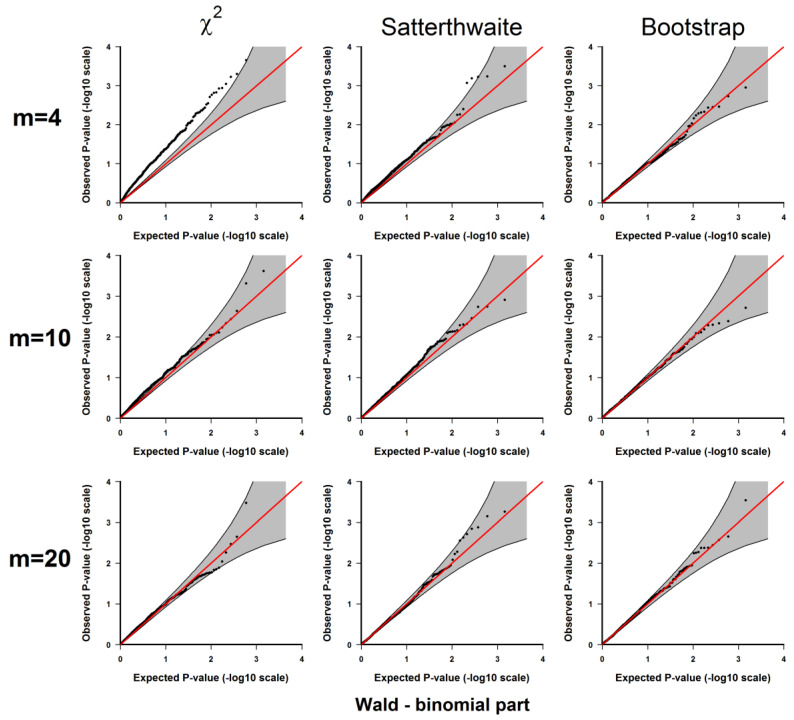
Results from 3.1.1 Evaluation of type I error rates. Plots of the observed versus the expected *p*-values for the Wald test for the binomial part under *H*_0_: no significant difference between the two conditions. The *p*-values are plotted on -log10 scale. The gray areas represent the 95% confidence interval bands of the expected *p*-values under *H*_0_.

**Figure A3:**
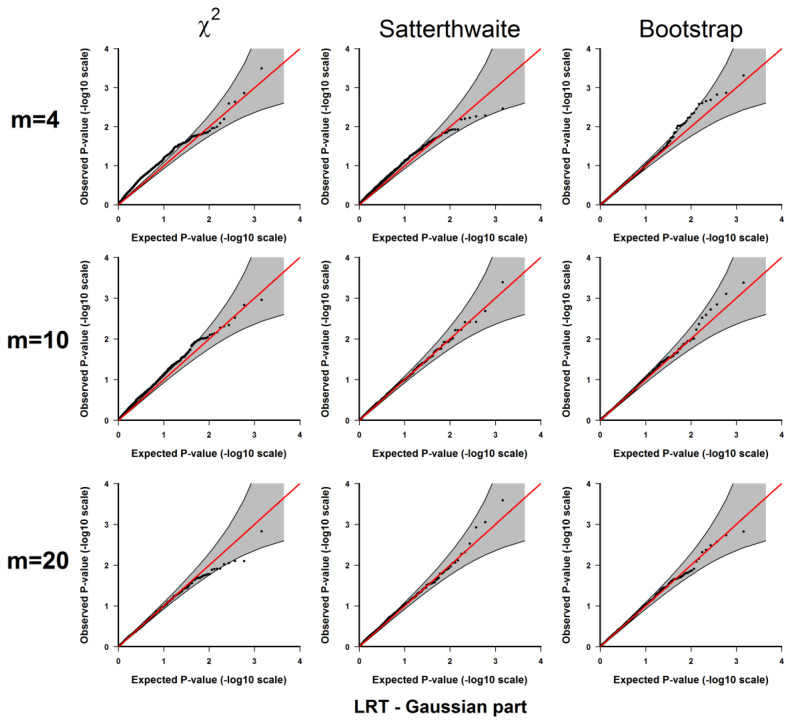
Results from 3.1.1 Evaluation of type I error rates. Plots of the observed versus the expected *p*-values for the likelihood ratio test for the Gaussian part under *H*_0_: no significant difference between the two conditions. The *p*-values are plotted on -log10 scale. The gray areas represent the 95% confidence interval bands of the expected *p*-values under *H*_0_.

**Figure A4:**
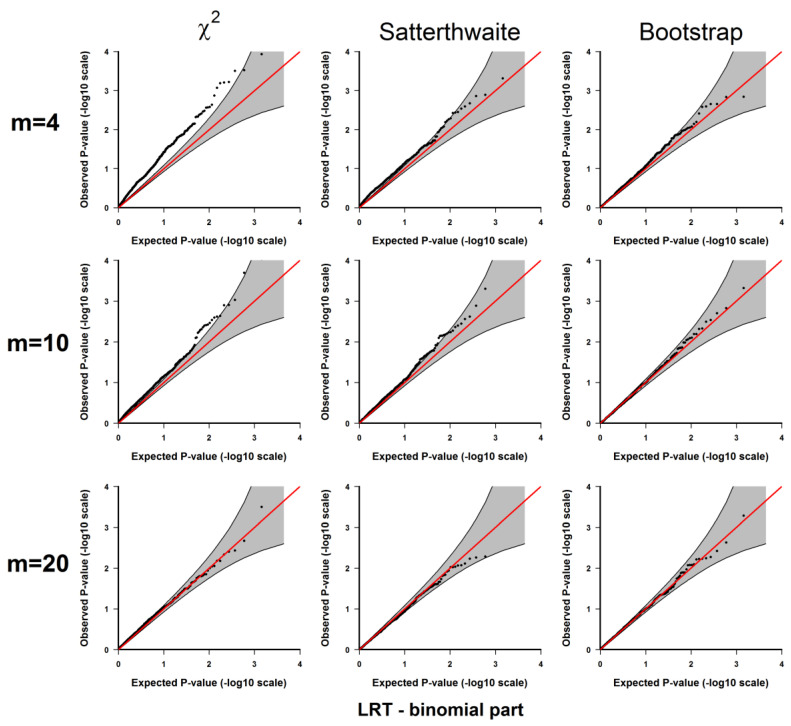
Results from 3.1.1 Evaluation of type I error rates. Plots of the observed versus the expected *p*-values for the likelihood ratio test for the binomial part under *H*_0_: no significant difference between the two conditions. The *p*-values are plotted on -log10 scale. The gray areas represent the 95% confidence interval bands of the expected *p*-values under *H*_0_.

**Figure A5:**
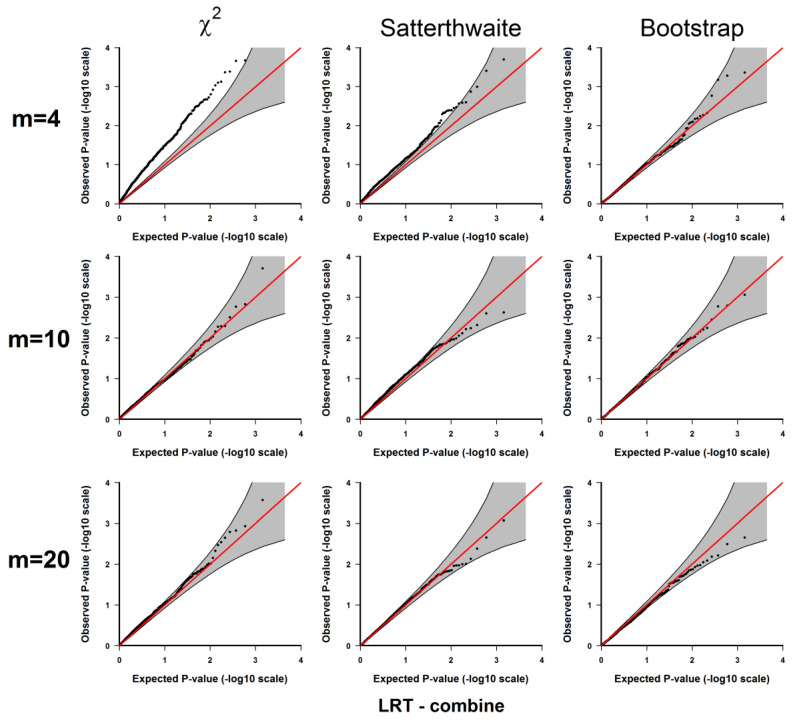
Results from 3.1.1 Evaluation of type I error rates. Plots of the observed versus the expected *p*-values for jointly testing the Gaussian and binomial parts under *H*_0_: no significant difference between the two conditions. The *p*-values are plotted on -log10 scale. The gray areas represent the 95% confidence interval bands of the expected *p*-values under *H*_0_.

If the type I error rate is correctly controlled, the *p*-values from the 1000 repetitions for each m should be uniformly distributed within 0 to 1, so we examine each method using the quantile-quantile plots of the above-calculated *p*-values from the simulated datasets (observed *p*-values) and the quantiles of uniform [0, 1] distribution (expected *p*-values), which are shown in Appendix A Figure A1, Figure A2, Figure A3, Figure A4 and Figure A5. As shown in these results, all 3 methods give well-controlled type I error rates for m=20. However, for small sample sizes (m=10 or m=4) the performance of controlling type I error rate of the 3 methods are ranked as (from the best to the worst): parametric bootstrap, Satterthwaite, the $\chi^{2}$ distribution. The inflation of the type I error rate is more severe for the $\chi^{2}$ distribution with the test for the binomial part (Figure A2 and Figure A4) or the joint test for the two parts (Figure A5). On the other hand, the parametric bootstrap takes the longest computational time, which can be overwhelming if we want to accurately estimate small *p*-values. As a general rule, if the sample size is large, then the $\chi^{2}$ distribution can be used. If the sample size is small, then the parametric bootstrap method should be preferred, even with the cost of longer computational time. The Satterthwaite method can be considered as an alternative method for a moderate sample size. Another strategy is to first use the *p*-values from the $\chi^{2}$ distribution or the rankings of the test statistics to identify those top differentially expressed genes and then use parametric bootstrap to further accurately estimate the *p*-values for those top genes.

#### 3.1.2. Evaluation of Statistical Power

In this section, we evaluate the statistical power of the TMM model and compare it with an existing method, MAST [12], and the two-part model with binomial and Gaussian parts but without random effects (named TM hereafter). The simulations are performed based on the following settings: suppose there are two biological conditions, and each condition has m/2 subjects, and for each subject i there are $n_{i}$ single-cell samples sequenced. To evaluate the power, we simulate the gene expression levels $y_{ijk}$ from the following model under $H_{1}$:

(11) $$\begin{array}{l} logit[\mathrm{Pr}(y_{ijk}=0)]=\log(\frac{\pi_{ijk}}{1-\pi_{ijk}})=\alpha_{1}+\alpha_{2}w+u_{i},\\ \log(y_{ijk}+1|y_{ijk}>0)=\beta_{1}+\beta_{2}x+v_{i}+e_{ij}, \end{array}$$

with $u_{i}\sim N(0,\sigma_{u}^{2})$, $v_{i}\sim N(0,\sigma_{v}^{2})$ and $e_{ijk}\sim N(0,\sigma_{e}^{2})$. In this model, w and x are 0/1 indicators of the conditions, and the effect sizes are represented by the parameters $\alpha_{2}$ and $\beta_{2}$, which correspond to the log odds of zero proportions and log fold change of the mean expression values for non-zero genes between the two conditions. The values of the parameters are set as follows: m=10, $n_{i}=20$ for all i’s (i=1,…,m), $\sigma_{u}=0.5$, $\sigma_{v}=1$, $\sigma_{e}=0.5$, $\alpha_{1}\sim N(0.25,0.25^{2})$, $\beta_{1}\sim N(3,0.5^{2})$. We then tune the effect sizes by varying $\left(\alpha_{2},\beta_{2}\right)$ for the following values: (0, 0), (0.25, 0.25), (0.5, 0.5), …, (1.5, 1.5). The simulations are repeated 1000 times for each different pairs of $\left(\alpha_{2},\beta_{2}\right)$’s. In each run, we apply our model TMM with the three methods for calculating *p*-values (the $\chi^{2}$ distribution, the Satterthwaite, and parametric bootstrap), MAST, and TM, respectively. The estimated power for each method is calculated as the proportion of *p*-values less than 0.05 among the 1000 repetitions.

**Figure 1:**
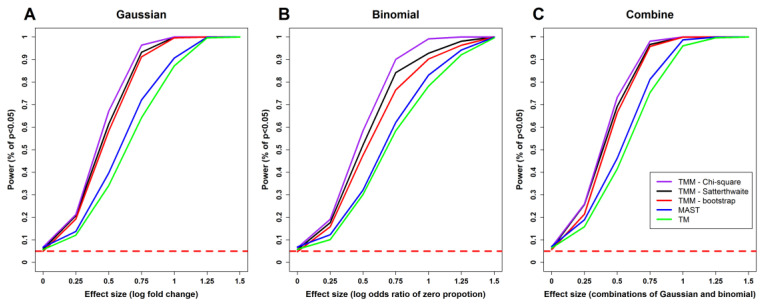
Comparisons of statistical powers of different methods. (**A**) Tests for the Gaussian part. (**B**) Tests for the binomial part. (**C**) Joint tests for the Gaussian and binomial parts. TMM: two-part mixed model. “Chi-square”, “Satterthwaite”, and “bootstrap”: the χ^2^ distribution, the Satterthwaite method, and parametric bootstrap method as described in Section 2.3. TM: the two-part model without random effects. The horizontal red dashed line represents the level of the test, which is α = 0.05.

Figure 1 shows the plots of power curves for each model with different effect sizes. As expected, the power of each method increases with effect size. The power of TMM is consistently higher than the other two models, which is also expected since we include random effects in this simulation setting.

## 4. Application to Real-World Single-Cell Gene Expression Data

### 4.1. Application to an scRT-qPCR Dataset

First, we apply the TMM model to an scRT-qPCR dataset and compare the results with MAST. This dataset is described in [23] and is incorporated with the MAST package [12], where 456 single-cell samples of T cells from 2 patients with human immunodeficiency virus (HIV) are isolated, and the expression levels of 75 genes related to the immune system function are measured by scRT-qPCR. The activation of two immune-response proteins, T cell receptor Vβ (TCR-Vβ) and CD154, are used to categorize those T cells, and the 456 single cells are divided into the following 4 different groups: TCR-Vβ+/CD154+, TCR-Vβ+/CD154−, TCR-Vβ−/CD154+, and TCR-Vβ−/CD154−, where the TCR-Vβ+ CD154+ group is the activated T cells with normal immune functions [23]. The goal of the analysis is to identify differentially expressed genes across the above four groups.

We fit MAST and our TMM model to this dataset. Specifically, the following two covariates are included in MAST:

$X_{1}$: a categorical variable indicating which of the above four groups the sample belongs to, where the TCR-Vβ+/CD154+ is coded as the reference group. This variable is the one of interest.

$X_{2}$: a categorical variable indicating which of the two subjects the sample is from.

For our TMM model, $X_{1}$ is included as a fixed effect in both the binomial part and the Gaussian part. The two subjects are treated as two clusters, which are included as random effects in TMM. The likelihood ratio test is used to test the individual Gaussian part and binomial part and also to jointly test the two parts, and the $\chi^{2}$ distribution approximation is used to calculate *p*-values for saving the computational time.

**Figure 2:**
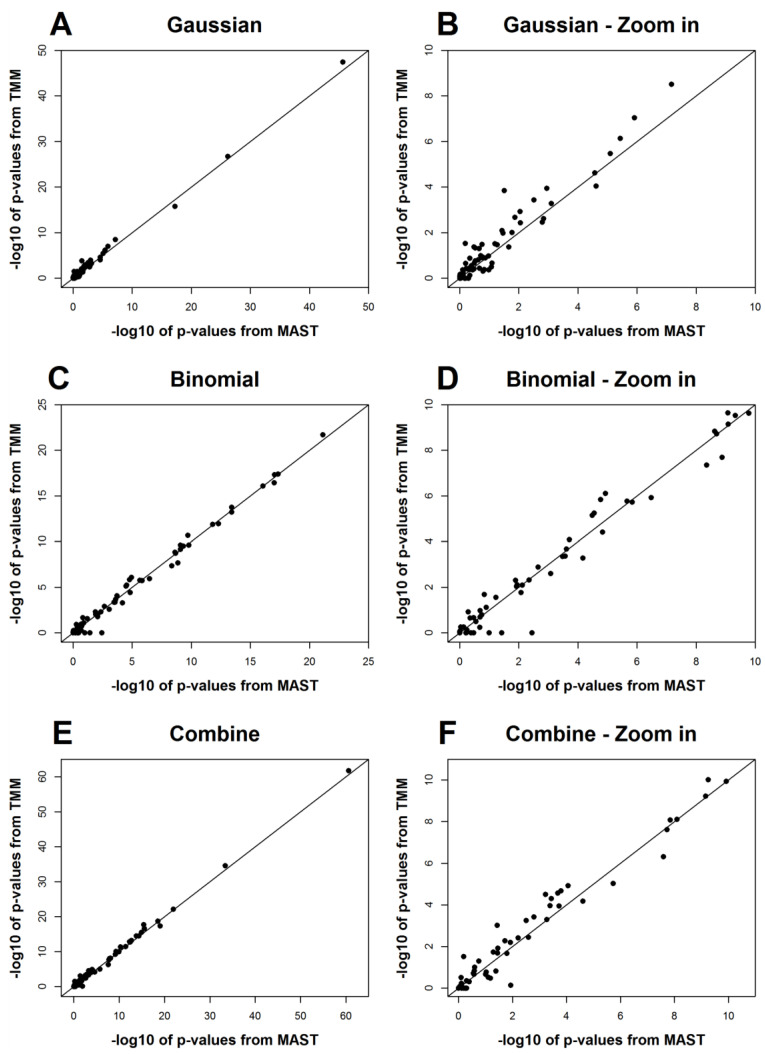
Comparisons of the *p*-values from TMM and MAST for the scRT-qPCR dataset. The -log 10 of the *p*-values from both methods are plotted. (**B**,**D**,**F**) are, respectively, the zoom-in parts of (**A**,**C**,**E**) on the range of 0 to 10.

The results from MAST and TMM for the 75 genes are shown in Table A1, and Figure 2 is a graphical comparison of the *p*-values from the two methods. We can see that the results from the two methods agree with each other in general, though some genes show different *p*-values from the tests for the zero-proportions (binomial part) (Figure 2). This is expected as there are only two clusters in this dataset, and the clustering effects do not play a significant role in this example. In fact, there should be a reasonable number of clusters included in a mixed effect model to make it useful in practice [15]. Therefore, MAST should be preferred for this dataset rather than TMM, and the application of TMM here is for the purpose of demonstration. On the other hand, these results show that TMM is not essentially worse than MAST, even if the clustering effects are not significant.

### 4.2. Application to scRNA-seq Datasets

A recent study compared various methods for differential expression analysis in scRNA-seq using a number of scRNA-seq datasets with matched bulk RNA-seq in the same purified cell types as reference [24]. This study showed that pseudobulk methods, which first aggregates reads across samples (i.e., biological replicates), transform a genes-by-cells matrix to a genes-by-samples matrix, and then uses methods for bulk RNA-seq such as DESeq [25], edgeR [26], and limma [27] for the following differential expression analysis, achieved the highest concordance with matched bulk RNA-seq results when the number of cells obtained from each sample is large (>500), while a negative binomial mixed model (NBMM) won when the number of cells per sample is not large (<200). Here we used one of those datasets containing both scRNA-seq and matched RNA-seq datasets made publicly available in [24], which was originally published in [28] to study the gene expression profile changes between five different types of CD4+ T cells stimulated by cytokines and unstimulated CD4+ T cells (control), to compare the performance of TMM with *p*-value evaluated by the empirical Satterthwaite method, an NBMM with the library size as an offset term implemented in [24] and a pseudobulk method using the likelihood ratio test in edgeR (referred as edgeR below).

**Figure 3:**
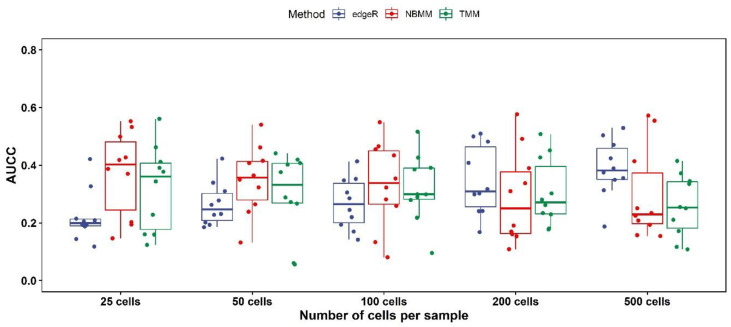
AUCC for TMM, NBMM, and edgeR in samples of between 25 and 500 cells from the CD4+ T cell data. The dots represent the AUCC values, and the boxplots represent their 75%, 50%, and 25 quantiles.

Following [24], we first obtain the lists of differentially expressed genes in the matched bulk data, and next apply the 3 aforementioned approaches for a series of downsampled scRNA-seq datasets, containing between 25 and 500 cells per sample from the original scRNA-seq datasets [28], and then calculate the area under the concordance curve (AUCC, ranges from 0 to 1 with 1 as perfect concordance and 0 as complete dissonance). The reason that we have to use the downsampled datasets is that the running time of NBMM is very long (see [24] and below), which prevents us from comparing these approaches to the full datasets. The results are shown in Figure 3, where we can see NBMM and TMM show higher concordance with matched bulk RNA-seq than edgeR when the number of cells per sample is not large (number of cells ≤ 200, Figure 3), while edgeR gives the highest concordance when the number of cells per sample is large (number of cells = 500, Figure 3). Regarding the running time: edgeR is the fastest with an average time of 1.7 min (including the time of the aggregating reads across samples); TMM has an average time of 53.2 min; NBMM is the slowest with an average time of 1174.3 min. These comparisons imply that TMM is more suitable for situations where the number of cells per sample is not large. We elaborate on these comparisons and the strengths of different approaches in the Section 5.

Next, we apply the TMM model to another scRNA-seq dataset and compare it with MAST and TM. This dataset is published in [7], which contains 466 single-cell samples from the human brain tissues of 8 adults (aged from 21 to 63 years) and 4 fetuses (all aged 16 to 18 weeks), and the expression levels of 22,088 genes in these samples are measured by scRNA-seq [7]. The dataset is available in NCBI Gene Expression Omnibus under accession number GSE67835.

The goal of our analysis is to identify differentially expressed genes between the adult and fetal brains. We fit TMM with the following two covariates as fixed effects:

$X_{1}$: a 0/1 indicator of biological conditions (adult versus fetus), which is the variable of interest;

$X_{2}$: the gender of the subjects: male and female for adults. The gender of the fetuses is coded as a third category, “undeveloped”.

The 12 subjects are treated as clusters, which are included as random effects in the model. The likelihood ratio test is used to test the individual Gaussian part and binomial part and also to jointly test the two parts, and the $\chi^{2}$ distribution approximation is used to calculate *p*-values for saving the computational time. We also fit the MAST and TM models, where *X*_1_ and *X*_2_ are included as covariates in these two models. Multiple comparison adjustment is performed using the Benjamini–Hochberg FDR procedure [29].

**Figure 4:**
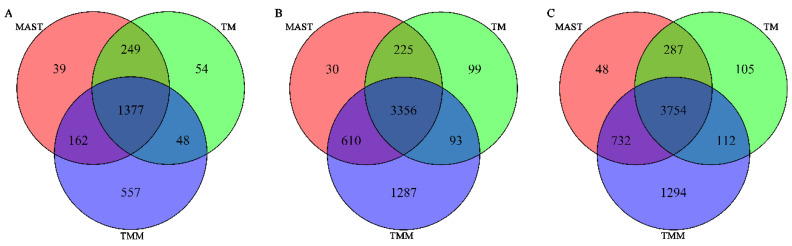
Number of differentially expressed genes identified by each method with FDR < 0.01. (**A**) Gaussian part. (**B**) Binomial part. (**C**) Joint test for the Gaussian and binomial parts.

Figure 4 shows the number of differentially expressed genes identified by each method with FDR < 0.01, and Table A2 shows the *p*-values and FDR for the top 20 differentially expressed genes (ranked by the *p*-values from the joint test for both the Gaussian and binomial parts under the TMM model). We can see the results from the three models show considerable overlaps (Figure 4), and the top differentially expressed genes all show very significant *p*-values and FDR from all methods. Notably, the total number of differentially expressed genes detected by TMM with FDR < 0.01 is much larger than the other two methods.

## 5. Discussion

In summary, we present a two-part mixed model (TMM) for differential expression analysis with single-cell gene expression data. This model not only adequately accounts for the distinct features of single-cell expression data, including extra zero expression values, high variability, and clustered design, but also provides the flexibility of adjusting for covariates. Since scRNA-seq is still a developing and growing technology, it brings more challenges in data analysis than bulk RNA-seq. These challenges can be technical (e.g., the number of samples in scRNA-seq is large, and the sequencing experiments are performed in different batches [30]), and also can be biological (e.g., the distinct features of the single-cell gene expression data, as discussed in the Introduction). Several more recent studies show that several confounding factors often present in scRNA-seq experiments, which can lead to biased results. These factors can also be categorized as technical factors that are related to the design of experiments, such as batch effects [30], or biological factors such as the detection rate of genes [12,30], gene lengths, and GC percent[30]. These confounding factors can be adjusted in TMM; however, planning a good study design for scRNA-seq experiments to reduce the confounding factors is a more fundamental task [30].

More recently, several new models and approaches have been proposed for the DE analysis on scRNA-seq data. As studied in [24] and Section 4.2, the pseudobulk method, which mimics the data format in bulk RNA-seq by aggregating reads across samples and generating a genes-by-samples matrix, enables the usage of well-maintained tools developed for bulk RNA-seq such as DESeq [25], edgeR [26], and limma [27] for the analysis in scRNAseq, and those methods are faster and show higher concordance with the DE results from bulk RNA-seq when the number of cells per sample is large, which can be achieved with current sequencing platforms. Alternatively, our approach, TMM, has the strength of being reliable when the number of cells per sample is not large (e.g., scRT-qPCR data and scRNA-seq data with smaller sample sizes and less cost) and providing a test for checking if the proportions of zero or lowly expressed genes are different between biological conditions. As future work, the computational speed and *p*-value estimation of TMM should be further optimized, which is also common for many mixed-effect models [24]. On a separate note, in [24] and Section 4.2, the DE genes in the matched bulk RNA-seq datasets that were used to check the consistency of those methods for scRNA-seq were also identified using DESeq, edgeR, and limma, which may lead to the bias towards the higher concordance given by those pseudobulk methods using the same three packages.

## Data Availability

The computer codes for reproducing the results in this paper is available online at: https://github.com/shilab2017/two_part_mixed_model (accessed on 21 December 2021).

## Appendix A

**Table A1:** Results of the gene differential expression analysis for the HIV scRT-qPCR dataset. The top gene CD40LG that codes CD154 protein is highlighted.

Gene Name	MAST			TMM		
	Gaussian	Binomial	Combine	Gaussian	Binomial	Combine
**CD40LG**	2.33 × 10^−46^	9.87 × 10^−18^	2.82 × 10^−61^	3.73 × 10^−48^	3.53 × 10^−17^	1.72 × 10^−62^
GAPDH	6.60 × 10^−27^	8.44 × 10^−10^	4.04 × 10^−34^	1.78 × 10^−27^	2.30 × 10^−10^	2.82 × 10^−35^
TNF	1.60 × 10^−03^	7.70 × 10^−22^	1.13 × 10^−22^	3.48 × 10^−03^	1.89 × 10^−22^	7.94 × 10^−23^
TGFB1	6.08 × 10^−18^	2.73 × 10^−04^	9.61 × 10^−20^	1.75 × 10^−16^	4.31 × 10^−04^	5.01 × 10^−18^
IL2	1.46 × 10^−03^	4.53 × 10^−18^	3.06 × 10^−19^	2.39 × 10^−03^	3.90 × 10^−18^	2.05 × 10^−19^
IL16	2.21 × 10^−01^	9.21×10^−18^	2.93 × 10^−16^	4.90 × 10^−02^	4.74 × 10^−18^	2.42 × 10^−17^
IL2Rg	6.80×10^−08^	1.97 × 10^−10^	3.72 × 10^−16^	3.11 × 10^−09^	2.08 × 10^−11^	2.06 × 10^−18^
CXCR4	7.89 × 10^−04^	3.94 × 10^−14^	1.33 × 10^−15^	5.20 × 10^−04^	1.71 × 10^−14^	3.51 × 10^−16^
CCR7	3.60 × 10^−01^	8.61 × 10^−17^	4.88 × 10^−15^	4.20 × 10^−01^	8.38 × 10^−17^	3.54 × 10^−15^
CD3d	3.69 × 10^−06^	1.67 × 10^−10^	1.65 × 10^−14^	7.22 × 10^−07^	2.33 × 10^−10^	3.61 × 10^−15^
IL2Ra	9.09 × 10^−03^	5.14 × 10^−13^	2.08 × 10^−13^	1.18 × 10^−03^	1.09 × 10^−12^	6.11 × 10^−14^
CD69	7.99 × 10^−06^	2.06 × 10^−09^	4.00 × 10^−13^	3.34 × 10^−06^	1.89 × 10^−09^	1.80 × 10^−13^
IL10	1.75 × 10^−01^	3.88 × 10^−14^	5.74 × 10^−13^	3.25 × 10^−02^	5.85 × 10^−14^	1.44 × 10^−13^
FASLG	6.47 × 10^−02^	1.60 × 10^−12^	3.73 × 10^−12^	3.06 × 10^−02^	1.33 × 10^−12^	3.32 × 10^−12^
IL7R	1.23 × 10^−06^	2.18 × 10^−06^	5.23 × 10^−11^	9.05 × 10^−08^	1.68 × 10^−06^	4.57 × 10^−12^
IL6ST	1.13 × 10^−03^	4.49 × 10^−09^	1.21 × 10^−10^	1.14 × 10^−04^	4.45 × 10^−08^	1.12 × 10^−10^
SLAMF1	3.45 × 10^−02^	4.78 × 10^−10^	5.56 × 10^−10^	1.05 × 10^−02^	2.95 × 10^−10^	9.47 × 10^−11^
IFNg	2.66 × 10^−05^	1.47 × 10^−06^	7.06 × 10^−10^	2.39 × 10^−05^	1.85 × 10^−06^	5.83 × 10^−10^
CD109	8.97 × 10^−01^	8.36 × 10^−10^	8.06 × 10^−09^	7.76 × 10^−01^	7.10 × 10^−10^	7.65 × 10^−09^
TNFRSF9	3.20 × 10^−01^	2.38 × 10^−09^	1.44 × 10^−08^	2.13 × 10^−01^	1.45 × 10^−09^	8.35 × 10^−09^
DPP4	2.96 × 10^−01^	1.35 × 10^−09^	1.91 × 10^−08^	4.66 × 10^−02^	2.03 × 10^−08^	2.38 × 10^−08^
ICOS	2.41 × 10^−05^	6.80 × 10^−05^	2.53 × 10^−08^	8.93 × 10^−05^	5.14 × 10^−04^	4.77 × 10^−07^
CD28	2.14 × 10^−01^	3.34 × 10^−07^	1.88 × 10^−06^	3.67 × 10^−01^	1.17 × 10^−06^	9.26 × 10^−06^
CD4	1.06 × 10^−01^	1.47 × 10^−05^	2.46 × 10^−05^	1.04 × 10^−01^	3.88 × 10^−05^	6.66 × 10^−05^
CD27	1.94 × 10^−01^	2.86 × 10^−05^	8.73 × 10^−05^	1.01 × 10^−01^	5.68 × 10^−06^	1.20 × 10^−05^
CD48	4.55 × 10^−01^	1.69 × 10^−05^	1.59 × 10^−04^	3.52 × 10^−01^	1.46 × 10^−06^	2.14 × 10^−05^
SLAMF5	5.39 × 10^−02^	3.28 × 10^−04^	1.89 × 10^−04^	3.34 × 10^−02^	4.43 × 10^−04^	1.14 × 10^−04^
CTSD	3.11 × 10^−03^	7.58 × 10^−03^	2.14 × 10^−04^	3.59 × 10^−04^	8.01 × 10^−03^	2.68 × 10^−05^
CD5	5.47 × 10^−01^	3.30 × 10^−05^	3.65 × 10^−04^	3.80 × 10^−01^	7.13 × 10^−06^	4.89 × 10^−05^
TBX21	1.71 × 10^−01^	1.97 × 10^−04^	4.06 × 10^−04^	1.17 × 10^−01^	8.15 × 10^−05^	1.10 × 10^−04^
CSF2	6.55 × 10^−01^	2.46 × 10^−04^	5.40 × 10^−04^	1.00	2.09 × 10^−04^	5.13 × 10^−04^
CD3g	9.95 × 10^−01^	1.17 × 10^−05^	6.03 × 10^−04^	8.13 × 10^−01^	7.87 × 10^−07^	3.11 × 10^−05^
TIA1	1.70 × 10^−02^	1.29 × 10^−02^	1.64 × 10^−03^	9.89 × 10^−03^	4.89 × 10^−03^	3.84 × 10^−04^
CD45	2.94 × 10^−01^	8.41 × 10^−04^	2.56 × 10^−03^	1.72 × 10^−01^	2.48 × 10^−03^	3.63 × 10^−03^
PECAM1	3.68 × 10^−02^	1.21 × 10^−02^	3.14 × 10^−03^	7.98 × 10^−03^	9.12 × 10^−03^	5.55 × 10^−04^
NT5E	5.96 × 10^−01^	2.21 × 10^−03^	6.24 × 10^−03^	3.77 × 10^−01^	1.28 × 10^−03^	3.82 × 10^−03^
LIF	7.70 × 10^−01^	3.60 × 10^−03^	1.17 × 10^−02^	6.83 × 10^−01^	1.00	7.22 × 10^−01^
FOXP3	8.77 × 10^−03^	2.03 × 10^−01^	1.21 × 10^−02^	3.65 × 10^−03^	2.02 × 10^−01^	6.33 × 10^−03^
TIMP1	2.18 × 10^−02^	1.26 × 10^−01^	1.62 × 10^−02^	4.23 × 10^−02^	7.55 × 10^−02^	2.13 × 10^−02^
CTLA4	3.26 × 10^−01^	8.50 × 10^−03^	1.92 × 10^−02^	4.15 × 10^−02^	1.70 × 10^−02^	5.31 × 10^−03^
FAS	7.88 × 10^−01^	4.45 × 10^−03^	3.49 × 10^−02^	4.21 × 10^−01^	4.76 × 10^−03^	1.21 × 10^−02^
RORC	8.99 × 10^−01^	1.13 × 10^−02^	3.61 × 10^−02^	9.52 × 10^−01^	7.71 × 10^−03^	2.06 × 10^−02^
CCR2	3.11 × 10^−02^	2.07 × 10^−01^	3.73 × 10^−02^	1.42 × 10^−04^	5.63 × 10^−01^	9.61 × 10^−04^
BCL2	2.34 × 10^−01^	3.77 × 10^−02^	4.15 × 10^−02^	1.56 × 10^−01^	1.00	1.51 × 10^−01^
PRDM1	1.36 × 10^−02^	5.71 × 10^−01^	5.18 × 10^−02^	2.11 × 10^−03^	7.40 × 10^−01^	1.85 × 10^−02^
CCL3	1.48 × 10^−01^	1.01 × 10^−01^	6.68 × 10^−02^	4.16 × 10^−01^	1.00	3.36 × 10^−01^
CCL2	8.07 × 10^−02^	1.00	8.07 × 10^−02^	2.14 × 10^−01^	1.00	3.05 × 10^−01^
IL8	1.03 × 10^−01^	2.06 × 10^−01^	9.36 × 10^−02^	4.31 × 10^−01^	1.07 × 10^−01^	1.67 × 10^−01^
CCL5	8.38 × 10^−02^	2.80 × 10^−01^	9.98 × 10^−02^	3.18 × 10^−01^	3.17 × 10^−01^	2.20 × 10^−01^
TNFSF10	3.27 × 10^−01^	1.49 × 10^−01^	1.76 × 10^−01^	3.88 × 10^−01^	2.06 × 10^−02^	5.12 × 10^−02^
CSF1	1.79 × 10^−01^	4.38 × 10^−01^	2.57 × 10^−01^	1.23 × 10^−01^	2.20 × 10^−01^	9.77 × 10^−02^
CCR4	4.57 × 10^−01^	1.79 × 10^−01^	2.66 × 10^−01^	4.10 × 10^−01^	1.59 × 10^−01^	1.48 × 10^−01^
HLADRA	1.61 × 10^−01^	5.11 × 10^−01^	2.73 × 10^−01^	4.78 × 10^−01^	1.21 × 10^−01^	2.16 × 10^−01^
BAX	9.26 × 10^−01^	5.98 × 10^−02^	2.86 × 10^−01^	9.97 × 10^−01^	2.79 × 10^−02^	1.94 × 10^−01^
CD38	4.60 × 10^−01^	3.35 × 10^−01^	4.09 × 10^−01^	7.37 × 10^−01^	2.11 × 10^−01^	4.97 × 10^−01^
SLAMF7	5.01 × 10^−01^	1.00	5.01 × 10^−01^	1.00	1.00	1.00
GATA3	1.36 × 10^−01^	9.75 × 10^−01^	5.11 × 10^−01^	1.29 × 10^−01^	8.29 × 10^−01^	4.44 × 10^−01^
PCNA	8.92 × 10^−01^	3.40 × 10^−01^	5.52 × 10^−01^	8.19 × 10^−01^	1.00	1.00
MMP9	6.35 × 10^−01^	1.00	6.35 × 10^−01^	1.00	1.00	1.00
ENTPD1	6.50 × 10^−01^	1.00	6.50 × 10^−01^	2.97 × 10^−02^	1.00	3.10 × 10^−02^
CCL4	6.79 × 10^−01^	6.22 × 10^−01^	7.55 × 10^−01^	1.00	1.00	1.00
PRF1	9.51 × 10^−01^	4.12 × 10^−01^	7.67 × 10^−01^	7.96 × 10^−01^	1.00	1.00
EOMES	7.68 × 10^−01^	5.98 × 10^−01^	7.89 × 10^−01^	5.52 × 10^−01^	1.00	7.63 × 10^−01^
IL6R	6.31 × 10^−01^	7.39 × 10^−01^	7.98 × 10^−01^	2.22 × 10^−01^	5.60 × 10^−01^	5.96 × 10^−01^
CCR5	4.52 × 10^−01^	9.28 × 10^−01^	8.09 × 10^−01^	1.33 × 10^−01^	5.54 × 10^−01^	3.07 × 10^−01^
GZMA	4.02 × 10^−01^	9.95 × 10^−01^	8.52 × 10^−01^	2.78 × 10^−01^	8.78 × 10^−01^	7.79 × 10^−01^
CD8a	9.58 × 10^−01^	1.00	9.58 × 10^−01^	6.68 × 10^−01^	1.00	9.00 × 10^−01^
B3GAT1	1.00	1.00	1.00	1.00	1.00	1.00
CXCL13	1.00	1.00	1.00	1.00	1.00	1.00
IL12RbII	1.00	1.00	1.00	1.00	1.00	1.00
IL13	1.00	1.00	1.00	1.00	1.00	1.00
IL22	1.00	1.00	1.00	1.00	1.00	1.00
IL3	1.00	1.00	1.00	1.00	1.00	1.00
IL4	1.00	1.00	1.00	1.00	1.00	1.00
MKI67	1.00	1.00	1.00	1.00	1.00	1.00

**Table A2:** *p*-values and FDR for the top 20 differentially expressed genes. The list of genes is ranked by the *p*-values from the combined test for both the Gaussian and binomial parts under the TMM model.

Gene Name	TMM						MAST					
	Gaussian		Binomial		Combine		Gaussian		Binomial		Combine	
	*p*-Value	FDR	*p*-Value	FDR	*p*-Value	FDR	*p*-Value	FDR	*p*-Value	FDR	*p*-Value	FDR
TMSB15A	2.14 × 10^−12^	5.31 × 10^−10^	1.60 × 10^−55^	3.33 × 10^−51^	1.57 × 10^−55^	3.27 × 10^−51^	4.50 × 10^−09^	4.66 × 10^−07^	1.42 × 10^−44^	3.01 × 10^−40^	1.35 × 10^−44^	2.86 × 10^−40^
MEX3A	2.01 × 10^−10^	2.66 × 10^−08^	1.55 × 10^−50^	1.62 × 10^−46^	1.34 × 10^−50^	1.39 × 10^−46^	3.73 × 10^−08^	2.79 × 10^−06^	1.57 × 10^−42^	1.67 × 10^−38^	1.57 × 10^−42^	1.67 × 10^−38^
SPARCL1	2.31 × 10^−15^	1.42 × 10^−12^	1.53 × 10^−49^	1.07 × 10^−45^	1.29 × 10^−49^	8.94 × 10^−46^	1.22 × 10^−13^	6.46 × 10^−11^	7.91 × 10^−38^	5.60 × 10^−34^	7.32 × 10^−38^	5.18 × 10^−34^
CLU	3.86 × 10^−14^	1.75 × 10^−11^	7.64 × 10^−43^	3.98 × 10^−39^	7.54 × 10^−43^	3.93 × 10^−39^	3.24 × 10^−12^	1.11 × 10^−09^	1.36 × 10^−33^	5.78 × 10^−30^	1.26 × 10^−33^	5.23 × 10^−30^
IL6ST	2.78 × 10^−06^	8.37 × 10^−05^	5.91 × 10^−42^	2.46 × 10^−38^	5.24 × 10^−42^	2.19 × 10^−38^	5.82 × 10^−04^	7.40 × 10^−03^	3.49 × 10^−33^	1.06 × 10^−29^	3.17 × 10^−33^	9.61 × 10^−30^
CRYAB	1.90 × 10^−13^	6.51 × 10^−11^	4.47 × 10^−39^	1.55 × 10^−35^	4.06 × 10^−39^	1.41 × 10^−35^	2.62 × 10^−11^	6.78 × 10^−09^	1.66 × 10^−34^	8.82 × 10^−31^	1.43 × 10^−34^	7.60 × 10^−31^
ALDOC	1.30 × 10^−16^	9.72 × 10^−14^	2.84 × 10^−36^	8.46 × 10^−33^	2.28 × 10^−36^	6.79 × 10^−33^	2.50 × 10^−14^	1.87 × 10^−11^	2.93 × 10^−29^	5.66 × 10^−26^	2.65 × 10^−29^	5.11 × 10^−26^
OSBPL1A	3.47 × 10^−20^	6.03 × 10^−17^	1.09 × 10^−35^	2.76 × 10^−32^	9.29 × 10^−36^	2.35 × 10^−32^	4.44 × 10^−20^	1.35 × 10^−16^	1.71 × 10^−33^	6.07 × 10^−30^	1.48 × 10^−33^	5.23 × 10^−30^
HTRA1	1.77 × 10^−13^	6.16 × 10^−11^	1.19 × 10^−35^	2.76 × 10^−32^	1.01 × 10^−35^	2.35 × 10^−32^	3.43 × 10^−11^	8.68 × 10^−09^	1.73 × 10^−27^	2.45 × 10^−24^	1.46 × 10^−27^	2.07 × 10^−24^
PRNP	6.70 × 10^−24^	3.50 × 10^−20^	2.33 × 10^−35^	4.87 × 10^−32^	2.24 × 10^−35^	4.66 × 10^−32^	5.61 × 10^−19^	1.32 × 10^−15^	4.92 × 10^−30^	1.16 × 10^−26^	4.04 × 10^−30^	9.53 × 10^−27^
TSPYL2	1.46 × 10^−19^	2.03 × 10^−16^	8.68 × 10^−35^	1.65 × 10^−31^	8.53 × 10^−35^	1.62 × 10^−31^	8.81 × 10^−19^	1.87 × 10^−15^	6.39 × 10^−29^	1.13 × 10^−25^	6.17 × 10^−29^	1.09 × 10^−25^
BHLHE41	3.97 × 10^−12^	9.01 × 10^−10^	1.08 × 10^−34^	1.88 × 10^−31^	9.52 × 10^−35^	1.66 × 10^−31^	3.60 × 10^−08^	2.70 × 10^−06^	6.76 × 10^−28^	1.03 × 10^−24^	6.45 × 10^−28^	9.79 × 10^−25^
CD24	4.01 × 10^−15^	2.39 × 10^−12^	1.51 × 10^−34^	2.43 × 10^−31^	1.37 × 10^−34^	2.19 × 10^−31^	9.82 × 10^−14^	5.35 × 10^−11^	1.09 × 10^−29^	2.32 × 10^−26^	9.27 × 10^−30^	1.97 × 10^−26^
NEUROD6	1.46 × 10^−12^	3.80 × 10^−10^	1.62 × 10^−32^	2.42 × 10^−29^	1.53 × 10^−32^	2.28 × 10^−29^	1.41 × 10^−10^	3.03 × 10^−08^	2.14 × 10^−30^	5.69 × 10^−27^	1.73 × 10^−30^	4.59 × 10^−27^
ADD3	7.02 × 10^−14^	2.87 × 10^−11^	1.18 × 10^−31^	1.64 × 10^−28^	9.83 × 10^−32^	1.37 × 10^−28^	5.81 × 10^−10^	9.23 × 10^−08^	2.65 × 10^−22^	1.94 × 10^−19^	2.37 × 10^−22^	1.68 × 10^−19^
BCL11A	5.72 × 10^−14^	2.44 × 10^−11^	2.92 × 10^−31^	3.81 × 10^−28^	2.42 × 10^−31^	3.16 × 10^−28^	1.32 × 10^−10^	2.90 × 10^−08^	1.44 × 10^−26^	1.80 × 10^−23^	1.16 × 10^−26^	1.45 × 10^−23^
SLC6A1	2.85 × 10^−17^	2.58 × 10^−14^	1.04 × 10^−30^	1.27 × 10^−27^	8.63 × 10^−31^	1.06 × 10^−27^	5.76 × 10^−14^	3.42 × 10^−11^	1.35 × 10^−21^	8.43 × 10^−19^	1.34 × 10^−21^	7.50 × 10^−19^
NR3C1	5.03 × 10^−07^	1.93 × 10^−05^	5.15 × 10^−30^	5.66 × 10^−27^	4.30 × 10^−30^	4.89 × 10^−27^	1.20 × 10^−09^	1.68 × 10^−07^	3.01 × 10^−27^	4.00 × 10^−24^	3.06 × 10^−27^	4.06 × 10^−24^
NEUROD2	2.34 × 10^−06^	7.27 × 10^−05^	4.56 × 10^−30^	5.29 × 10^−27^	4.45 × 10^−30^	4.89 × 10^−27^	7.12 × 10^−06^	2.22 × 10^−04^	3.74 × 10^−28^	6.11 × 10^−25^	3.68 × 10^−28^	6.01 × 10^−25^
ALCAM	5.90 × 10^−15^	3.33 × 10^−12^	6.98 × 10^−30^	7.28 × 10^−27^	5.98 × 10^−30^	6.24 × 10^−27^	1.80 × 10^−16^	2.25 × 10^−13^	2.25 × 10^−23^	1.99 × 10^−20^	2.13 × 10^−23^	1.81 × 10^−20^
